# Climbing robots for manufacturing

**DOI:** 10.1093/nsr/nwad042

**Published:** 2023-02-20

**Authors:** Bo Tao, Zeyu Gong, Han Ding

**Affiliations:** State Key Laboratory of Digital Manufacturing Equipment and Technology, School of Mechanical Science and Engineering, Huazhong University of Science and Technology, Wuhan 430074, China; State Key Laboratory of Digital Manufacturing Equipment and Technology, School of Mechanical Science and Engineering, Huazhong University of Science and Technology, Wuhan 430074, China; State Key Laboratory of Digital Manufacturing Equipment and Technology, School of Mechanical Science and Engineering, Huazhong University of Science and Technology, Wuhan 430074, China

**Keywords:** climbing manufacturing robot, climbing robot, robotized intelligent manufacturing

## Abstract

Robotized intelligent manufacturing is a growing trend in the manufacturing of large and complex components in aviation, aerospace, marine engineering and other industries. With their expansive workspaces and flexible deployment, climbing manufacturing robots can create a revolutionary manufacturing paradigm for large and complex components. This paper defines the climbing manufacturing robot based on the application status of climbing robots and then analyzes four key technical requirements: adhesion, locomotion, localization and control. Subsequently, the current research status of climbing robots in these four areas is classified and reviewed, along with a clarification of the research frontiers and trends in each area, and the applicability of the relevant research to manufacturing-oriented climbing robotic systems is analyzed. Finally, by concluding the development trends of robotized intelligent manufacturing equipment in terms of manufacturing dimension and scale, environmental adaptability and cluster collaboration capability, we clarify the major challenges for climbing manufacturing robots in terms of adhesion principles, motion mechanisms, positioning technology and control methods, and propose future research directions in these fields.

## Teaser

This review analyzes the current state and development trends of climbing manufacturing robots, and anticipates their significant contributions to the paradigm revolution of manufacturing large and complex components.

## INTRODUCTION

Manufacturing is the cornerstone of human civilization. High-end equipment in aviation, aerospace, ocean engineering and other fields is the crystallization of human technology. With the advancement of science and technology, the sizes of aircraft skins, ship shells and other large components are increasing, reaching tens of metres or even hundreds of metres. Structures are being developed with asymmetric free-form surfaces that vary in thickness. Service performance requirements are increasing, thus posing harsh requirements for manufacturing quality. The modern manufacturing paradigm centralized with CNC machine tools is limited by the spindle stroke and cannot satisfy the efficiency requirement of *in-situ* manufacturing. Manual operation still plays an essential role in the manufacture of large and complex components and is inefficient and poor in quality. Therefore, achieving high-performance manufacturing and maintenance of the entire lifecycle of large components is a significant challenge for manufacturing science.

Robotized intelligent manufacturing is the current trend in the manufacture of large and complex components, sparking worldwide interest. The United States established the Advanced Robotics Manufacturing Institute [1] in 2017 to develop innovative applications of robotics in advanced manufacturing fields. Since 2010, the EU has been funding three major projects—COMET [2], HEPHESTOS [3] and MEGAROB [4]—to develop ‘plug-and-produce’ robotic machining systems for high-precision, hard-material and large-size parts. The Natural Science Foundation of China launched the Tri-Co Robot Major Research Program in 2016, which aims at the creation of ‘craftsmen’ robots for intelligent manufacturing of large and complex components [5]. With the support of the Tri-CO Robot Program, Chinese universities, represented by Tsinghua University, Tianjin University, Huazhong University of Science and Technology, and Shanghai Jiao Tong University, have studied the optimized design method of machining robots [6,7], proposed approaches to ensure the accuracy of robotic machining [8,9], developed machining robots with high precision and high performance, and realized the robotized milling, welding, grinding and polishing of large and complex components [10–12].

The main benefit of robotized intelligent manufacturing of large components is their ability to circumvent operational space constraints by integrating various mobile operating robotic platforms. Such mobile capabilities expand the operational range of robots. However, insufficient reachability and a lack of environmental adaptation are ongoing issues with the robotized manufacturing paradigm because of the severe lack of vertical movement space-covering capabilities. For instance, large airplanes can reach heights of more than 10 m, whereas the working height of industrial robots is often less than 4 m. Another example is the in-service maintenance of large ships, where the hull is tens of metres high and needs to be maintained in water and air media. Clearly, given these working conditions, mobile manipulators cannot realize *in-situ* manufacturing.

Climbing robots are special robots that cruise vertical structures using different adsorption methods and locomotion mechanisms. They are highly adaptable to the environment because of their unique form of motion: as long as they can attach and move perfectly on the surface of a physical entity, climbing robots will, theoretically, have an unrestricted motion space. Thus, climbing robots have unparalleled deployment flexibilities. In fact, it will come as a surprise that climbing robots are particularly positioned to overcome the reachability bottleneck in robotized intelligent manufacturing when considered in the context of large component manufacturing demand. Based on climbing robots equipped with different actuators, such as grinders, drills, or inspection and maintenance equipment, *in-situ* machining and in-service full-cycle maintenance of large components can be achieved.

In view of this situation, this paper first defines climbing manufacturing robots on the basis of current applications and clarifies the four key capabilities needed. Subsequently, the research status of adhesion, locomotion, localization and control is reviewed in detail. Finally, the challenges and trends in climbing manufacturing robots are discussed.

## DEFINITION OF CLIMBING MANUFACTURING ROBOTS

### Current status of climbing robot applications

**Figure 1.**
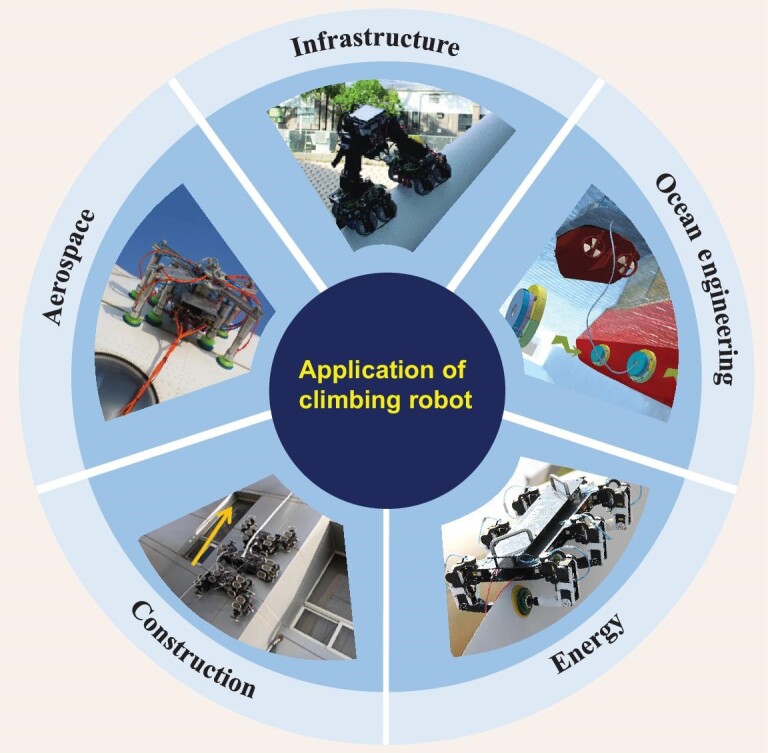
The current application status of climbing robots. Climbing robots were applied in a variety of fields, such as aerospace, ocean engineering, infrastructure, energy and construction. Adapted with permission from Refs [14,24,26,36,49], respectively.

Climbing robots have been successfully used for inspection, maintenance and manufacturing tasks in various fields, as shown in Fig. 1.

In the field of aerospace, the application of climbing robots is focused on completing tasks in the manufacturing and maintenance of aircraft or tasks, such as the exploration of extreme environments. For example, under the guidance of a laser tracker, a negative-pressure climbing robot performed precise hole-making operations and in-service inspection of the wing [13]. The inspection of aircraft skins has been performed by a variety of climbing robots, such as legged negative pressure adsorption and vortex [14–16]. In outer space, a quadrupedal climbing robot can explore extreme terrains, such as cliffs [17]. A gecko-inspired grasping system can manipulate uncooperative objects in a microgravity environment [18].

In the ocean engineering field, because the environment has significant cross-media characteristics, the applications of climbing robots can be divided into two categories. The first category focuses on ship manufacturing processes such as welding, inspection and cleaning. Such applications usually employ magnetic adsorption for surface attachment with diverse locomotion mechanisms, such as crawlers [19,20], differential wheels [21] or novel wheels [22]. The second category is the underwater inspection and maintenance of ships in service. Negative pressure adsorption and bionic adhesion are preferred considering the underwater environment. Many novel adsorption techniques have been proposed, such as the amphibious adsorption mechanism that mimics suckerfish [23] and noncontact negative pressure adsorption based on Bernoulli's principle [24].

In the field of energy, the application of climbing robots focuses on two scenarios. The first scenario involves the inspection and maintenance of facilities for energy production. Such scenarios are common in fields in which manual work is dangerous. For example, a multi-robot cooperative inspection system was deployed for the daily inspection and maintenance of wind power towers and blades [25]. Climbing robots for this task already have mature commercial products, such as the blade bug robot funded by the UK’s Multi-platform Inspection, Maintenance and Repair in Extreme Environments project and the BR-8 robot funded by the Wind Turbine Repair Robot project of the EU’s Horizon 2020 program [26,27]. Another application is the non-destructive inspection of pipelines, where climbing robots based on magnetic adhesion are used to inspect the inside [28,29] and outside of a pipeline [30–32].

Construction is one of the most mature areas for climbing robot applications, particularly the cleaning and inspection tasks of skyscrapers. To cope with the complex façade structures and non-smooth surface of buildings, different climbing robots have been proposed, such as negative pressure adsorption using bipedal inchworm-type locomotion mechanisms [33,34] and the crawler mechanism [35,36]. In addition, a novel negative-pressure adsorption unit was proposed to handle the rough walls of buildings [37–39]. Another typical class is rope-based climbing motion on the building façade. A series of rope-winch–based climbing robots was proposed for façade cleaning [40–42]. In addition, building surfaces can be inspected and cleaned by a flying-climbing composite robot based on propeller mechanisms [43,44].

Infrastructure facilities, such as bridges, require tedious inspection and maintenance, where climbing robots can serve as an important supplement to human inspection. One of the most common applications is the inspection of cable-stayed bridges, which can be performed using the split-type wire-driven cable-climbing robot CCRobot and its successor, which is equipped with flying capabilities [45,46]. To automatically repair the protective layer of cables, a cable-climbing robot based on quadrilateral independent suspension is proposed [47]. In addition, different forms of climbing robots using magnetic adhesion with plane-crossing ability are proposed to inspect the steel structure of bridge bodies [48,49].

As can be observed, deploying climbing robots to replace manual inspection of hazardous areas is the most widespread application in climbing robot research. Despite their successful applications, climbing robots generally focus on simple tasks with low loads, such as inspection and cleaning. Current climbing robots are rarely directly involved in core manufacturing processes, such as machining and assembly; thus, the advantages offered by their unique operation patterns are not maximized. The manufacture of large components using climbing robots remains a research frontier worthy of further investigation.

### Definition of climbing manufacturing robots

The climbing manufacturing robot is a subbranch of climbing robots that seamlessly combines climbing robotics with manufacturing needs. Several representative climbing manufacturing robots have emerged recently. For instance, Huazhong University of Science and Technology developed a climbing machining robot for *in-situ* manufacturing of large and complex components. By designing flexible negative pressure adsorption chambers and the corresponding active compliant adsorption method, the robot adsorbs up to 1000 N with a self-weight of 10 kg and can adapt to variable curvature surfaces with a radius of curvature greater than 1.1 m. A global positioning method has also been proposed for precise localization in a wide range of large components [50,51]. Tsinghua University also developed a novel flexible climbing machining robot for processing large components. It consists of two serially connected parallel manipulators: one for adsorption and the other for 5-axis machining. With systematic stiffness evaluation and optimization, the robot is promising for achieving efficient and economical processing of large-scale structural parts [52].

In summary, to serve as a carrier platform integrated with specific manufacturing actuators, the climbing manufacturing robot should have the following features compared with ordinary climbing robots: (1) they can be stably adsorbed on the manufacturing surface in different manufacturing environments, such as machining or maintenance, and overcome the effects of dynamic loads, such as machining forces; (2) they can move flexibly on the surface to be manufactured according to craft requirements; (3) they can sense their own positions in a wide range with the accuracy required to meet the manufacturing level; and (4) they can realize autonomous control with the manufacturing crafts as constraints.

From the above definitions, the following technical issues must be addressed for climbing manufacturing robots:

#### Adhesion

Adhesion capability is an essential feature of climbing robots. However, for climbing manufacturing robots, the adhesion capability should be more concerned with adaptability to the manufacturing object and different environments, such as rugged surfaces, wet surfaces, or surfaces with variable curvatures. For machining applications, such as milling and grinding, the adhesion should be sufficiently strong to overcome the machining force. Adaptability to dirty and dynamically changing machining environments is another concern for adhesion technology.

#### Locomotion

Traditional climbing robots have various locomotion mechanisms, such as wheels, leg-feet, and crawlers. Similar to the adhesion requirements, environmental adaptability for manufacturing tasks is the first priority for the climbing manufacturing robot's locomotion module. The selected locomotion mechanism should be capable of adapting to curved surfaces and should have the corresponding obstacle-crossing capability on the surface to be manufactured. Movement efficiency is also particularly important in facilitating manufacturing efficiency.

#### Localization

Climbing manufacturing robots should be able to accomplish precise positioning in indoor or outdoor settings, with different types of references in differing quantities. In this regard, the positioning tasks must first involve absolute positioning, which entails creating a global coordinate system in accordance with the manufacturing task and tracking the robot's position within this coordinate system in real time. Moreover, it should be possible to track the relative position of the robot with respect to the particular feature to be manufactured to meet the machining and assembly requirements.

#### Control

Manual teleoperation is insufficient for the efficient manufacturing of large components. Autonomous control is an important prerequisite for robotized intelligent manufacturing. The control issue of climbing manufacturing robots should focus on two aspects: autonomous motion planning according to the structural features and craft requirements of the manufacturing object and accurate and robust motion control on the curved surface of large components.

The development of climbing robots in terms of the four major issues described above is reviewed in this paper, and the gap between current technological advancements and the demand for robotized intelligent manufacturing is addressed.

## ADHESION PRINCIPLES FOR CLIMBING ROBOTS

**Figure 2.**
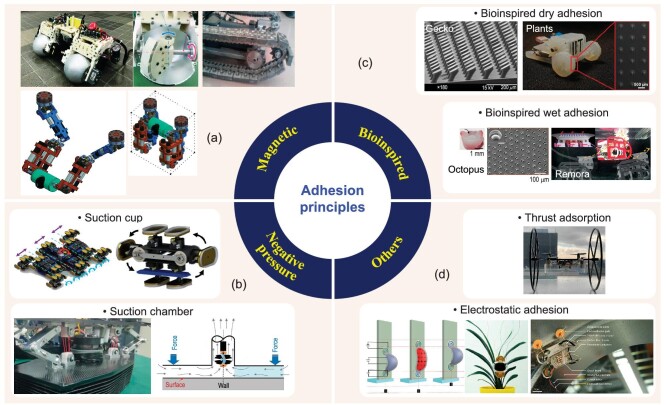
Adhesion principles for climbing robots. (a) Magnetic adhesion integrated with wheels, tracks and legs. Adapted with permission from Refs [22,20,54], respectively. (b) Negative pressure adsorption, which can be divided into suction cup based and suction chamber based. Adapted with permission from Refs [36,50,59], respectively. (c) Bioinspired adhesion, which can be divided into bioinspired dry adhesion and bioinspired wet adhesion. Adapted with permission from Refs [64,72,74,78], respectively. (d) Other adhesion methods, whose representatives include thrust adsorption and electrostatic adhesion. Adapted with permission from Refs [44,88,89], respectively.

Adhesion ability directly determines the operational capability of the climbing robot. The mainstream adhesion methods include magnetic adhesion, negative pressure adsorption and bionic adhesion. The different types of adhesion principles for climbing robots are shown in Fig. 2.

### Magnetic adhesion

Magnetic adhesion is the most mature adhesion method used for climbing robots. A permanent magnet or electromagnetic magnet can be employed to achieve adsorption on ferromagnetic surfaces. Tracks are the most common motion mechanism for magnetic adsorption robots. For example, the tracked robot proposed in [20] combines magnetic caterpillars and can climb over uneven ferromagnetic surfaces with a speed of up to 7 m/min for vertical structure inspection in shipbuilding. In addition, multi-body tracked magnetic climbing robots can be formed, and transition between different planes can be realized by coordinating different single robots [53]. The reciprocating mechanism and roller chains can be integrated with a magnetic track to enable adaptation to different structural shapes with coated or noncoated steel surfaces and the transition between different surfaces [48]. The arm-like structure can also be combined with magnetic wheels, thus enabling walking on steel structures spanning different planes [49]. The magnetic adsorption unit can also be directly mounted on a robotic arm to achieve a more compact magnetic multimodal climbing manipulator [54]. The magnetic unit can also be separated from the locomotion mechanism. In the omnidirectional magnetic wheeled climbing robot used for steel pipe inspection, instead of being integrated, a permanent magnetic unit is installed next to the wheels. The wheel can comply with the pipe surface using the magnetic adsorption force along with the suspension [30,31]. Innovative work has been conducted on adsorption actuators, such as ball-shaped magnetic wheels with two degrees of freedom (DOFs) of rotation and suspension [22], which can align the magnetic force with the normal direction of the surface.

Magnetic adsorption is ideal for machining or maintenance on ferromagnetic surfaces because it can provide a strong yet stable adsorption force. However, it is unable to adsorb lightweight nonferromagnetic materials that are widely used in aerospace and other fields, thus greatly limiting the scope of its application.

### Negative pressure adsorption

Generating the atmospheric pressure or hydraulic pressure difference between the internal and external parts of the adsorption chambers or suction cups is the essence of the negative pressure adsorption method. Therefore, in accordance with the source of the pressure difference, negative-pressure adsorption can be divided into two categories.

The first type is based on the vacuum pump that is outside the robot body and is connected to suction cups via an air tube. The suction cup is usually flexible and small and can be used for intermittent motion by rapid adsorption/desorption. Hence, suction cups are typically combined with legged or crawler locomotion mechanisms. Guan *et al*. proposed a biped climbing robot capable of transferring between different planes, whose suction module consists of suction cups integrated on the end with autonomous pose detection and alignment [33,34]. Inchworm-inspired [55] and Hirudinea-inspired [56] soft robots perform adsorption using two suction cups at the end driven by vacuum pumps. For tracked climbing robots, multiple suction cups on the track are usually automatically activated or deactivated by mechanical valves as the track rotates, whereas the steering is based on an independent steering suction cup [36]. It should be noted that the load/weight ratio of the robot based on suction cups and vacuum pumps is not satisfactory. For example, the weight of the robot in [36] is 70 kg, but its load is only 15 kg. The low load/weight ratio limits its application in machining tasks because the cutting force of machining is up to hundreds of newtons. Larger robots are needed if better load capability is required, thus compromising the flexibility of the deployment of the climbing robot and its adaptability to the environment. Moreover, the reliability of the adsorption system will decrease rapidly as the complexity of the pneumatic system increases with the number of suction cups.

Another type of negative-pressure adsorption uses centrifugal fans as vacuum sources, which are integrated into climbing robots by direct connection to the suction chambers via short air paths. The area of the suction chambers connected to the centrifugal fan is typically much larger than that of the suction cups, thus providing a stronger adsorption force. This setup is suitable for high adsorption force/weight ratio requirements and is a better choice for machining tasks. In our previous work [50], by developing flexible suction chambers with actuators with three degrees of freedom, along with the active compliance adsorption method, the climbing robot can achieve an adsorption force over 1000 N, with a self-weight of 10 kg. Similar to a vacuum pump, a centrifugal fan can provide negative pressure to multiple suction chambers [57]. The adsorption performance can be improved by improving the centrifugal fan's impeller [58]. Centrifugal fan-based adsorption can be used in aquatic environments. For example, an underwater climbing robot based on a sliding negative-pressure adsorption mechanism is proposed in [59], which can generate a maximum adsorption force of 600 kgf even at a gap height of 12 mm. Underwater adsorption can also be realized using contrarotating propellers based on the Bernoulli negative-pressure generation mechanism [24,60].

New principles of negative pressure generation are also a hot research topic in this field. One of the most representative methods is centrifugal force–based sealing, which exploits the rotational inertia effect of fluids to generate and maintain a negative pressure. With the use of a high-speed impeller to drive the fluids in the mechanism to rotate, a zero-pressure difference is formed on the edge of the suction chamber by the centrifugal force of the rotating fluids [37–39]. This novel mechanism can adsorb on coarse walls with a much lower frictional resistance because it is a noncontact adsorption mechanism. A similar principle is proposed for adsorption in an underwater environment by injecting high-pressure water inside a vortex suction cup via two axisymmetric side-distributed inlets to create a negative pressure area in the centre [61].

In short, negative-pressure adsorption can provide a stable and strong adsorption force and is easy to control. Moreover, it has few restrictions on the surface material. Hence, negative-pressure adsorption is a vital method for climbing manufacturing robots and has been applied in aircraft manufacturing [13], inspection [15], ship maintenance [24] and other fields.

### Bioinspired adhesion

Bioinspired adhesion is an active research topic in the field of climbing robots. Various creatures with adhesion abilities provide us with a wealth of inspiration for robot design. Currently, bioinspired adhesion research focuses on exploring various materials and processes to mimic, combine, or even surpass the biological adhesion mechanism. This paper classifies bioinspired adhesion into two major categories according to the application environment: bioinspired dry adhesive and bioinspired wet adhesion.

#### Bioinspired dry adhesive structure

The strong adhesion capacity of terrestrial creatures such as geckos, beetles, and spiders originates from micro- or nanoscale hairy structures at the contact interface.

Gecko-inspired adhesion is the most extensively studied type of bioinspired adhesion, and its essence is dry adhesion based on van der Waals forces. Numerous studies have explored mimicking the micro functional structures of the toe of a gecko. A necessary first step is to characterize the microstructural features and then explore different materials and processes to achieve reliable preparation. The most common materials used for gecko-inspired adhesives are synthetic polymers such as PDMS [62] and polyvinylsiloxane [63]. The fibre is usually a mushroom-shaped structure, forming a certain inclination angle to achieve directional adhesion and rapid desorption. This microfibre can be prepared from spun polyurethane film by photolithography and moulding combined with a constant load during the curing process [64]. In addition to mimicking the static characteristics of gecko feet, we can further analyze the internal structure and kinematic characteristics of gecko feet to improve adhesion ability. The muscles on the gecko's toes work in concert with their adhesive structures. This mechanism can be mimicked by generating a substrate with adjustable stiffness (such as thermoplastic polyurethane) on the lower layer of a mushroom-like fibre, whose elastic modulus can be modulated to enhance adhesion [65]. The substrate of the integrated adhesion layer can be attached to the robot foot using a flexible material to mimic the tendon, thus allowing the load to be distributed uniformly to enhance the adhesion capacity for large-area contact [66].

In addition to polymers, bionic microfibres can also be made from other materials. For example, randomly entangled adhesion layers can be grown on top of vertical single-walled carbon nanotubes to substantially increase shear adhesion while maintaining a normal adhesion constant [67]. This strong adhesion in the shear direction and easy detachment in the normal direction are promising for climbing robots (e.g. enhancing the friction of the robot locomotion mechanism to prevent slippage). The tip of the microfibre can have a shape other than mushroom-shaped, such as a microwedge [18]. This form of adhesion layer can work reliably in microgravity environments, such as space.

Another research trend in gecko-inspired adhesives is the modification of the microfibre structure to surpass its original performance. The development of deep learning methods provides new ideas for the morphological design of microfibres. Different shapes of microfibres can be randomly generated, and their adhesion performance is predicted by a neural network prediction model to select the shape with the optimal adhesion performance [68]. In addition, special adhesion capabilities can be obtained by fusing the microfibre structure with adhesion patterns of other organisms. For example, mussel-inspired special adhesion polymers can be spin-coated onto fibre arrays to achieve reliable underwater adhesion [69].

In addition to geckos, numerous insects capable of climbing have also inspired the design of climbing robots. Some insects use the micro-spine structure of their feet to achieve adhesion. This structure can be simulated by embedding flexible nitinol micro-spine structures in high-friction rubber on the wheels of climbing robots. The micro-spine endows the wheels with good adaptability to smooth, rough, or even wet surfaces [70]. A similar micro-spine mechanism can be fabricated by mimicking the adhesion structure of the inchworm and realizing reliable adhesion on different media [71].

Plants with wall-climbing abilities also inspire the design of adhesion structures for climbing robots. For example, the herbaceous climbing plant *Galium aparine* L mechanically interconnects the host surface with its microhooks. The microhook structure can be fabricated using a 3D direct laser lithography process with multiphoton adsorption, thus improving the mobility of the wheeled climbing robot [72]. The liana *Parthenocissus tricuspidata* attaches by means of tendrils and terminal sticky discs. This feature can be mimicked by polystyrene honeycomb-like microstructures fabricated using a novel hierarchical anodic aluminium oxide template. Underwater adhesion is achieved based on van der Waals and capillary forces generated by the close contact between the macro porous arrays and water molecules [73]. However, few robots achieve adhesion purely by using plant bionic structures. Plant-inspired adhesion can complement the adhesion capability and improve motion performance.

In general, the frontier of bioinspired dry adhesion research still lies in the fabrication process of various microstructures and the enhancement of the adhesion function, which are mostly in the laboratory stage with rare application in the practical tasks of climbing robots. Although the existing fabricated adhesion structures have limited durability and the fabrication processes are complex, it is undeniable that many organisms to be simulated have many excellent properties, making them a great potential for many machining applications. For example, the controllable strong adhesion properties of the dry adhesion of geckoes are particularly suitable for anchoring robots in machining operations such as drilling or milling.

#### Bioinspired wet adhesive structure

Numerous organisms that live in water or wet environments also possess outstanding adhesion abilities, such as octopi, snails, and tree frogs. However, wet adhesion is not exclusive to aquatic organisms. In principle, the adhesion of some terrestrial animals, such as some insects, also depends on the presence of liquid at the adhesion interface. Thus, it can also be classified as wet adhesion.

The octopus adhesion structure is representative of underwater adhesion and has a certain amphibious adhesion capacity. The tentacle sucker of the octopus evolved into a delicate structure. Simple micro-suction cup structures can be fabricated using perforated silicon moulds and polyurethane-acrylate prepolymers to achieve micro-concave structures [74]. The infundibulum of the suction cup has wrinkles that can enhance the adhesion performance. Wrinkles can be obtained by a PDMS adhesive layer surface subjected to silicone treatment. Adhesion under wet conditions can be significantly increased because of the enhanced drainage and capillary interaction between the wrinkles and bonding substrates [75]. In addition to polymeric materials such as PDMS, a variety of novel materials have recently been successfully applied in the fabrication of octopus-like suction cups, such as programmable organohydrogels with multi-stable mechanical states [76]. In fact, the adsorption of octopi is closely related to the structure of the octopus arms. The taper angle of the tapered soft actuator of the bionic octopus arm causes changes in the bending curvature and bending force, leading to differences in flexibility and grasping force [77]. Vacuum-driven suction cups can be integrated into the tapered octopus arm, which substantially improves adsorption performance.

Although the current bionic octopus suction cup has a good adsorption ability in air and liquid, it is still relatively rarely applied in climbing robots. The main difficulty is achieving efficient switching between adsorption and desorption. Moreover, its soft structure has natural defects in terms of durability and rigidity, limiting its application in machining tasks, which are very sensitive to precision and force.

In addition to the octopus, the remora, also known as suckerfish, is another representative aquatic creature with underwater adhesion ability. Its adhesion capability originates from its complex and sophisticated suction disc, which can be divided into three main parts. The active deformation of the lamella part creates a pressure difference between the inside and outside of the suction cup, and the lip part maintains the sealing of the contact area, whereas the spinule part composed of tiny hairs enhances friction. Accordingly, bionic suction discs with carbon nanotube micro-spine structures can be fabricated using replica 3D printing and micro- and nano-laser etching. With a bionic suction disc, the robot can produce an adhesion force 340 times the weight of the robot [78]. An aerial-aquatic hitchhiking robot combined with an improved bionic suction disc was designed to rapidly attach and separate on a variety of complex surfaces in air and underwater [23]. The properties of the clingfish adhesion structure render it suitable for climbing manufacturing robots. Simultaneous reinforcement of adsorption and friction is an urgent demand for applications in which large machining forces exist.

Many molluscs, such as snails, mussels and oysters, achieve adhesion with secreted mucus. Methods to mimic mucilaginous materials include pH-based modulation of stitching polymers [79] and sequence-controlled hydrogels with diverse cationic/aromatic monomers [80]. A similar bilayer structure can be inspired by slugs, which can achieve high adhesion energy on wet surfaces [81]. Despite its excellent adsorption properties, the mucus-based adhesion approach is not suitable for manufacturing oriented climbing robots because the presence of mucus is highly likely to violate the manufacturing process specifications.

Tree frogs are representative organisms with amphibious adhesion capability. The geometric patterns and mechanical properties of the keratinized epithelium on the tree frog's sticky toe pad, which can enhance adhesion and friction, can be mimicked by adhesion arrays consisting of PDMS micropillars embedded in polystyrene nanopillars [82].

The adhesion of some insects also relies on the principle of wet adhesion, which relies on the intermolecular, capillary, and van der Waals forces of the liquid film between the contact surfaces to realize adhesion. Wet adhesion pads with semi-elliptical hollow structures can be fabricated using a combination of electroforming and soft lithography, which has been successfully applied in hexapod robots [83]. Porous fibrous adhesion pads can also be designed to mimic insect contact splitting and wet adhesion principles [84].

In general, the application of bioinspired wet adhesion in climbing robots is rare in the manufacturing field because they are primarily made of soft or even liquid materials, thus posing difficulty in achieving standards for accuracy and rigidity in manufacturing operations. Existing fabrication methods are intricate and unaffordable for a wide range of applications. Nevertheless, the affinity of wet adhesion to aquatic environments makes it suitable for amphibious operations. Therefore, it has great application prospects in robot machining, inspection, and maintenance in the field of ocean engineering.

### Other adhesion methods

Thrust adsorption is the result of an intricate fusion of rotorcraft and climbing robot locomotion principles. The robot is pressed against curved surfaces using the thrust produced by the propeller and then moves it with certain locomotion mechanisms (usually wheels). For example, a climbing robot combined with a quadrotor with a quad chassis can generate a large frictional force for locomotion on steep slopes [85]. A similar mechanism using two wheels can be found in [44]. By connecting the wheels and propellers with the pulleys, the robot can maintain its flying capability as a regular quadcopter, aside from its climbing capability [86]. The weak thrust force of the propellers cannot provide a sufficient adsorption force for machining, whose cutting force is up to hundreds of newtons. Hence, climbing robots that use this adsorption method are typically used for inspection.

Electrostatic adhesion is a cutting-edge adsorption technology for climbing robots, the basic principle of which is to use an electrostatic attractive force. A high voltage is usually artificially applied to obtain sufficiently large electrostatic attraction. In recent years, electrostatic adhesion has been frequently reported in soft robots or micro-scale robots with different materials, such as borate ester polymer hydrogels that can rapidly switch between adhesive and non-adhesive states in response to a mild electrical stimulus [87]. Different electrostatic adhesion mechanisms can be used in climbing robots. For example, dielectric-elastomer artificial muscles combined with electro-adhesive feet can form a soft climbing robot that can perform spatiotemporally controlled adhesion, crawling and turning [88]. Similar electro-adhesive pads can be integrated into the leg structure with passive alignment ankles to achieve high-speed locomotion at a relatively low adhesion voltage [89]. Interdigital circular electrodes that generate induced charges can be integrated into small flying insects to achieve rapid adhesion, hovering and desorption [90]. Electrostatic adhesion is primarily constrained by its high-voltage requirement, which makes regulation challenging. It cannot yet be used for climbing machining jobs because its adhesion strength is still weak under existing technical conditions. However, its delicate adhesion principle makes it easy to apply to miniaturized climbing robots for inspection and maintenance in extreme environments or confined spaces.

## LOCOMOTION MECHANISMS FOR CLIMBING ROBOTS

**Figure 3.**
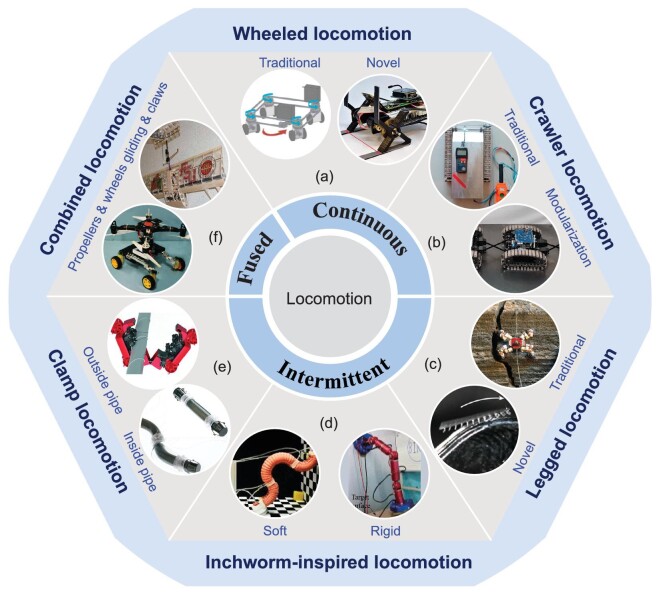
Locomotion mechanisms for climbing robots. For continuous locomotion, (a) wheeled locomotion has traditional and novel mechanisms. Adapted with permission from Refs [24,92], respectively. (b) Crawler locomotion can be based on traditional tracks or composed of modular systems. Adapted with permission from Refs [20,53], respectively. For intermittent locomotion, (c) legged locomotion has both traditional and novel multilegged mechanisms. Adapted with permission from Refs [17,97], respectively. (d) Inchworm-inspired locomotion is mainly based on soft or rigid mechanisms. Adapted with permission from Refs [33,55], respectively. (e) Clamp locomotion is divided into two categories based on whether the motion is inside or outside the pipe. Adapted with permission from Refs [46,103], respectively. In addition, (f) some climbing robots have combined locomotion mechanisms. Adapted with permission from Refs [85,106], respectively.

The locomotion mechanism for climbing robots can be divided into two categories according to their operational characteristics: continuous locomotion mechanism and intermittent locomotion mechanism. In continuous locomotion mechanisms, each module is in continuous contact with the adsorbed surface during motion. Examples of this category include wheeled and crawler locomotion. For intermittent locomotion mechanisms, each module exerts discontinuous contact with the adsorbed surface. Typical intermittent locomotion mechanisms include legged, inchworm-inspired, or clamp styles. In addition, there are climbing robots with locomotion mechanisms that use the fusion of multiple structures. Figure 3 depicts the various typical locomotion mechanisms of climbing robots.

### Continuous locomotion mechanism

#### Wheeled locomotion

Wheeled locomotion is well suited for climbing manufacturing robots because the surfaces of large components are typically continuous and freeform. A straightforward yet tested kinematic model with excellent movement efficiency presents an urgent demand for climbing manufacturing robots.

Research on traditional wheeled mechanisms has concentrated primarily on enhancing their ability to adapt to curved surfaces and overcome slippage. For instance, dual-wheel modules were created to balance forces on surfaces with varying curvatures and increase friction [24]. The spring plate connection between the wheels can isolate the additional force generated by the tyre mass when the suction module vibrates [91]. Adding two DOFs to magnetic adhesion wheels can achieve shape-adaptive capabilities while guaranteeing that the adhesion force is always directed normally to the surface [22].

Novel wheels have been developed that can be adapted to various unique working situations. Inspired by the ratchet-like attachment mechanism of the hook-climber *Galium aparine*, a novel micropatterned flexible wheel that can climb on rough surfaces with a 60° inclination was developed [72]. A wheg mechanism was developed to address the difficulties of plane-to-plane transitions and overcome barriers. It consisted of four X-shaped plates on which a three-layer flexible adhesion plate was integrated [92]. Similarly, a wheel with a climbing action profile can incorporate a gecko-inspired structure using adhesive tape [63].

As the most practical motion mechanism for climbing manufacturing robots, the wheeled mechanism still needs to solve the transition motion problem on discontinuous surfaces and resolve external interference, such as slippage and vibration under the influence of the machining force.

#### Crawler locomotion

Terrain adaptability and the capacity to navigate obstacles are exceptional characteristics of tracked and crawler locomotion mechanisms. Such abilities are easily integrated with various adhesion modules, such as magnetic [20] and negative pressure modules [35,36]. The frontiers in this field lie in the inventive design of tracked mechanisms and creative combinations of climbing robot architecture. Multiple sets of elastomeric tracks with adhesive capabilities can be connected by flexible joints and separately distributed on a climbing robot to simultaneously achieve compliant adhesion and flexible movement [93]. The tracked climbing robot may be modular and capable of reconfiguring. The modular tracked climbing robot joined using a particular connecting mechanism can actively apply torque to increase the contact force between the tracks and climbing surface for smoother surface transitions and obstacle crossing [53].

For machining applications, the crawler mechanism should concentrate on resolving the contradiction between adsorption force and environmental adaptability. For instance, to produce a strong adsorption force, tracked climbing robots often use magnetic adsorption, which places demands on the adsorption surface materials. For negative pressure tracks, multiple fine suction cups must be controlled, which have a limited adsorption force and, consequently, complex control requirements.

### Intermittent locomotion mechanism

#### Legged locomotion

Legged motion is another typical mode of locomotion for climbing robots, which is less effective but more environmentally adaptable than wheeled motions. In particular, the legged locomotion mechanism is suitable for challenging outdoor conditions, making it highly promising for climbing manufacturing.

The leg mechanism of a climbing robot is not fundamentally different from that of a traditional quadruped or multilegged robot. The typical idea is to integrate hook claws, micro-spines, or bionic adhesion structures on legs to enable climbing on a rough surface or in a field environment. Integrating claws that resemble grappling hooks into an elastic steel plate would allow legged climbing with passive compliance on rough walls [94]. A similar climbing motion can be realized by integrating fishing-hook–based grippers on a legged mechanism [95]. The leg of the quadrupedal robot LEMUR3 with micro-spin grippers is powered serially by seven actuators with redundant DOF [17], while the legs of the quadrupedal climbing robot realizing the lizard's gait are composed of four-bar linkages [96]. Certain novel legged locomotion mechanisms have been studied and used in various fields. For example, a climbing robot equipped with microscopic magnetic artificial cilia can climb on 0°–180° slopes either above or submerged in water with a carrying capacity greater than 10 times its own weight [97]. Smebot, a soft-footed climbing robot, has a unique modular design. By integrating different foot modules (including electromagenetic, suction, dry adhesion) onto a soft body driven by shape memory alloy wires, it can climb on different surfaces [98].

Legged climbing robots have a low load capacity on vertical surfaces owing to the stiffness of their locomotion mechanisms. Thus, they are generally used for inspection. One of the frontier problems for climbing manufacturing robots is increasing their load capacity while retaining the strong environmental adaptability of legged structures.

#### Clamp locomotion

The locomotion mechanisms for pipe climbing can be inspired by animal climbing and bird perching movements. In accordance with whether they are utilized inside the pipe or outside, these mechanisms can be classified into two categories.

The movement outside the pipe is typically accomplished via the clamping principle, which entails gripping the pipe's exterior and using friction to achieve axial motion along the pipe. For example, a suspension system with springs can be used to connect V-shaped rollers to clamp the rollers to a steel cable to achieve climbing [47]. Clamping can also be attained by two semicircular structures hinged together with four palms inside the circle, while the motion along the rope or pipe is driven by ducted fans [46]. Soft robotics and bionic robotics have also provided novel concepts for robot clamping. For instance, the branch holding motion can be accomplished by a caterpillar-inspired soft robot with a silicone inverted mould body and a finned gripping mechanism [99], as well as by an arboreal snake-inspired winding climbing robot with two winding actuators and one telescoping actuator [100].

The research focus of robots that acquire climbing motion inside pipes lies in the delicate and soft locomotion mechanism design, owing to their limited motion space. For instance, crawling motion through pipes can be achieved using vacuum-powered silicone rubber muscles based on the origami principle [101]. This can also be accomplished by designing two distinct air chambers that can either support the inner wall of the pipe or telescopically bend to move backwards and forwards using a similar origami concept [102]. The inspection of pipelines with sub-centimetre diameters and different curvatures can also be realized by a smart material–driven pipeline inspection robot, which consists of dielectric elastomer actuators as artificial muscles and smart composite microstructure–based anchoring units as transmissions [103].

Although climbing robots with clamping locomotion mechanisms have only been reported in inspection applications, clamping climbing is a viable locomotion pattern given the prevalence of tubular constructions in daily life, along with increasing machining and maintenance demands.

#### Inchworm-inspired locomotion

Inchworm-inspired locomotion uses an arm-like structure with several DOFs to move by alternating adsorption and desorption on both ends of the arm. They can be divided into two categories according to the stiffness of the arm-like structure.

Typically, multi-joint robotic arms are used to create a rigid inchworm-inspired climbing robot, which is represented by the climbing robot created by Guan Yishen *et al*. [33,34,104], which has a rigid five-jointed structure. Similarly, symmetrical 6 DOF robotic arms can be used to endow a climbing robot with the ability to cross obstacles [49]. Cutting-edge inchworm-inspired mechanisms focus on soft-body swinging structures and their driving methods. Multimodal motion switching between crawling and climbing for a soft inchworm-inspired climbing robot can be achieved using different pneumatic artificial muscles [55]. The crawling motion can be driven by a dielectric elastomer actuator stimulated by a voltage to bend and extend [88]. In addition, when a constant air pressure is transformed into a time-varying undulating oscillation force on the buckling sheet, the buckling sheet ring oscillator can generate a climbing motion [105].

Overall, although soft inchworm-like motion mechanisms with low loading capacities and weak motility are currently impractical for machining, their flexible style of motion and strong environmental adaptation still suggest wide-ranging opportunities for climbing manufacturing robots.

### Combined locomotion

Each locomotion mechanism has its own drawbacks. However, combining several mechanisms makes it possible to concurrently benefit from environmental adaptation, load-carrying and motion efficiency.

Considering that the climbing robot needs to climb on a vertical surface, combining different types of flying mechanisms with the climbing mechanism can dramatically increase the robots’ environmental adaptability. For example, a pair of claws and fixed wings can be combined to create a robot that can glide and climb vertical walls [106]. A climbing robot based on propeller thrust and a portable rope can utilize propellers and a rope ascender to achieve adsorption and climbing [40]. Similarly, the climbing robot can press against and climb on a steep surface using propellers with two additional DOFs [85]. In fact, the wheels can be motorless in the propeller-wheel mechanism because the attitude and driving force can be achieved simultaneously with sufficient propellers [43]. Otherwise, a pulley can be used to drive both the propellers and wheels simultaneously to achieve the climbing mode [86].

Combined locomotion mechanisms for climbing robots are ideal for inspections in a variety of hazardous locations. For heavy-duty manufacturing activities, such as milling or drilling, they still need to increase their load capacity.

## LOCALIZATION TECHNOLOGIES FOR CLIMBING ROBOTS

The majority of current research on climbing robots is focused on the realization of climbing functions. Comparatively speaking, localization has received less attention because early climbing robots are typically controlled by manual observation and teleoperation. However, accurate localization is a prerequisite for ensuring the quality of machining tasks that have high requirements for geometric precision and surface quality. This section reviews localization techniques that are already in use for climbing robots and explores potential techniques for climbing manufacturing.

**Figure 4.**
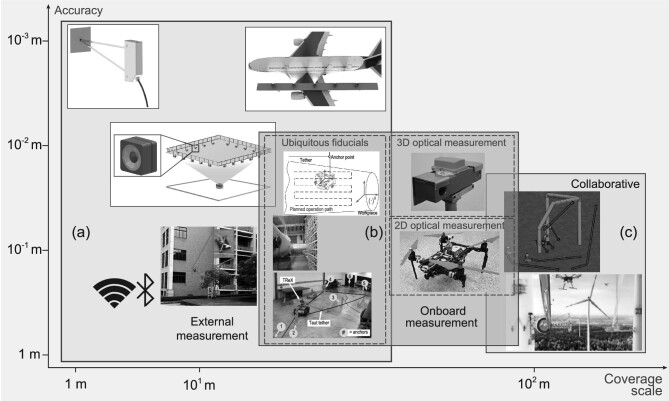
Accessible localization methods for climbing robots. (a) Localization based on external measurements, can be realized by high precision laser tracker or laser distance sensor, external camera or camera array, or wireless communication. Adapted with permission from Ref. [107]. (b) Localization based on onboard measurement, can be divided into localization using 2D optical measurement, localization based on 3D optical measurement and localization based on ubiquitous fiducials. Adapted with permission from Refs [51,108,113,119], respectively. (c) Multirobot Collaborative Localization, can be realized by employing different kinds of robots. Adapted with permission from Refs [25,32], respectively.

According to information sources, the localization methods of climbing robots can be divided into methods based on external measurements, onboard measurements, and multirobot collaborative localization. These three categories and their typical performance are shown in Fig. 4. The overlapping areas in Fig. 4 imply that two adjacent methods are identical or similar in principle.

### Localization based on external measurements

The basic idea behind localization based on external measurements is to acquire global robot poses using external measurement equipment. For instance, visual measurements utilize an arranged global camera to obtain poses for climbing robots [107]. However, a single global camera measurement is insufficient to satisfy the manufacturing requirements. In the industrial field, a more common approach is to use high-precision equipment such as laser trackers. The BAE Corp. uses a Leica laser to obtain the climbing robot's location by tracking a target that was set on the robot. The expanded Kalman filter method combines data from numerous sensors, achieves precise robot position estimation, and offers tool point precision for aircraft manufacturing tolerances [13]. Positioning methods based on external mobile wireless sensor networks are also promising. For example, an ultrasonic-based beacon system with fixed beacons interlinked via radio waves can be used to obtain the precise location of the climbing robot [21].

The aforementioned external equipment-based localization approaches often have a conflict between accuracy, range and cost. Specifically, a visual approach based on a single camera has a constrained field of vision, making it challenging to ensure accuracy from a distance. In addition, mounting external cameras is difficult owing to the unique working environment of climbing robots. A laser tracker system has excellent precision and extensive coverage; however, it is inoperable when the laser is blocked. The adsorbed surfaces for climbing robots on large components are typically complex curved surfaces, in which case the laser of a single station is susceptible to obstruction when the robots arrive at various positions. The high expense of multi-station laser tracker systems, such as iGPS, will unavoidably limit industrial applications.

### Localization based on onboard measurements

For field operations, such as in-service maintenance of large-scale equipment, climbing robots can rely on onboard sensors only to perform wide-range localization if the environment is too hostile for external measuring equipment. Achieving self-localization by fusing information without the need for external measurement is similar to the goal of SLAM. As a result, extensive SLAM research can serve as a helpful reference for the localization of climbing robots.

#### Combined localization based on 2D optical sensors

In a variety of inspection tasks, the most popular configuration is a climbing robot with cameras, which also provides the hardware foundation for localization. Localization methods that combine visual data with additional sensors have been successfully implemented in robotics. VINS-Mono [108], which uses a single camera and a low-cost inertial measurement unit, has a robust procedure for estimator initialization and loop detection, thus making it suitable for field climbing robots when executing inspection or machining tasks *in situ*. In fact, many similar localization methods have the potential to be applied in climbing robots for manufacturing, such as feature-based monocular ORB-SLAM [109]. Evidently, this feature-based approach can be useful for localizing climbing robots on surfaces with numerous textural features. For scenes that lack textural features, direct sparse odometry, which is independent of keypoint detectors or descriptors, can be used [110]. In addition to traditional camera imaging, optical flow sensors can be used to obtain two-dimensional (2D) relative displacement information, which can be combined with an accelerometer to realize track reckoning in wheeled climbing robots [111].

Overall, the combined localization method based on 2D image information has a low hardware cost, good robustness and adaptability, which is beneficial for climbing manufacturing robots. However, the absolute positioning accuracy at long distances still needs to be improved to meet the manufacturing requirements, especially for machining, such as hole making or milling.

#### Combined localization based on 3D optical sensors

LiDAR can obtain 3D point cloud information of the environment. Compared with the traditional 2D image, although the point cloud information is relatively sparse, it provides the 3D relative position natively, which is more convenient for estimating the climbing robot's pose on vertical surfaces. For example, LiDAR scan registrations combined with wheel encoders and accelerometers can be used to position climbing robots in 3D pipe structures [29]. In the oil and gas environment, with the computer-aided design model as a prior, 6 DOF localization of a robot can be achieved based on a real-time point cloud acquired by LiDAR using a time-efficient likelihood function and particle filter framework [112]. Curved 3D surfaces can be modelled from noisy LiDAR point clouds using a tool named tensor voting [28]. The point cloud can also be acquired using stereo cameras and RGB-D cameras [113]. The RGB-D images and IMU measurements can be fused to generate pose-coupled key frames with depth information to realize self-localization for the climbing robot and further enable the visualization of surface inspection [114].

Considering the 3D motion features of the climbing robot, cutting-edge 3D SLAM can be utilized. For example, a map of a large 3D environment can be represented by an evidence-based grid–based volumetric submap [115]. This strategy can be used for the localization of climbing robots in field operation scenarios, such as in-service inspection and maintenance of large wind turbine blades. For an outdoor environment with complex yet conspicuous profile features, localization can be realized by integrating robust visual odometry with real-time 3D mapping methods by generating consistent global models annotated with semantic labels [116].

3D visual measurement-based localization offers inherent advantages in climbing robot position estimation. However, existing studies have paid limited attention to accuracy, which is not sufficient to meet the demands of robotized machining.

#### Localization based on ubiquitous fiducials

Ubiquitous fiducial-based localization means that the climbing robot uses naturally existing or artificially arranged fiducials in the environment to achieve self-localization. This category is somewhat similar to the method based on external measurement, as indicated in Fig. 4, with an overlapping region of *Onboard Measurement* and *External Measurement*. The essential difference lies in the installation position of the measurement equipment. In the method based on ubiquitous fiducials, the measuring equipment is mounted on the robot, whereas the observed fiducial is in the environment. Meanwhile, because the onboard sensor might be cameras, the ubiquitous fiducial-based localization is also similar to the aforementioned optical sensing-based methods. The essential difference is whether the entire environment must be reconstructed to achieve self-localization. The method based on ubiquitous fiducials only achieves self-localization and does not consider environmental reconstruction.

The use of vision sensors to observe distinct visual features to obtain the robot's own pose with high accuracy is a practical idea that has been widely used in various inspection tasks. Artificial fiducials were verified in our previous study, in which the mobile robotic system was used to manufacture large components [117,118]. The localization and navigation of UAVs based on visual coded fiducials have been extensively studied; thus, visual fiducials, such as coded markers, may also provide reliable information for the positioning of climbing robots.

Another novel fiducial-based method is the use of ropes for localization. Typically, equipment operating at a certain height must be equipped with safety ropes to ensure security. With the ubiquity of ropes for climbing robots and their measurable lengths, their anchor points can be regarded as fiducials. In the pioneering work of tethered simultaneous localization and mapping, the pose of the robot and the positions of the anchor points are estimated by measuring the length and bearing of the deployed tether [119]. The error introduced by the elasticity of the rope and slippage of the pulley should be addressed further [41]. In our previous study, a tether displacement sensor was introduced to acquire the global pose. Visual inertial odometry with reduced drift was proposed by introducing adsorption constraints [51]. Despite the ingenuity and accessibility of the rope-based climbing robot localization method, its accuracy is typically insufficient for manufacturing applications. To further increase localization accuracy, future studies may consider the combination of rope information with visual and inertial data.

### Multirobot collaborative localization

The manufacturing of large and complex components requires a robot cluster to work collaboratively owing to its large size. This condition leads to another challenge, namely, collaborative localization among multiple robots.

Related research has been conducted on the collaborative localization of multiple climbing robots. Tavakoli *et al*. proposed a collaborative localization scheme for pipe-climbing robots and ground mobile robots. Ground mobile robots are equipped with cameras to reconstruct the environment and act as mobile observers for pipe-climbing robots using visual markers. They tracked and guided the climbing robot by measuring its pose and the structure being climbed [32]. A multirobot system composed of climbing robots and multicopters for automated wind turbine maintenance was proposed in [25]. Multicopters with vision and LiDAR sensors are used to guide slow-climbing maintenance robots and for global inspection.

Collaborative localization is foreseen to lie at the core of cluster manufacturing by multiple climbing robots and relies on the localization method based on onboard measurement, as indicated in Fig. 4. Research on how to realize robust communication, efficient data management, and effective information sharing among climbing robots is important [120]. At the same time, complementary positioning mechanisms between climbing robots are also fundamental questions that are worth exploring.

## CONTROL METHOD FOR CLIMBING ROBOTS

**Figure 5.**
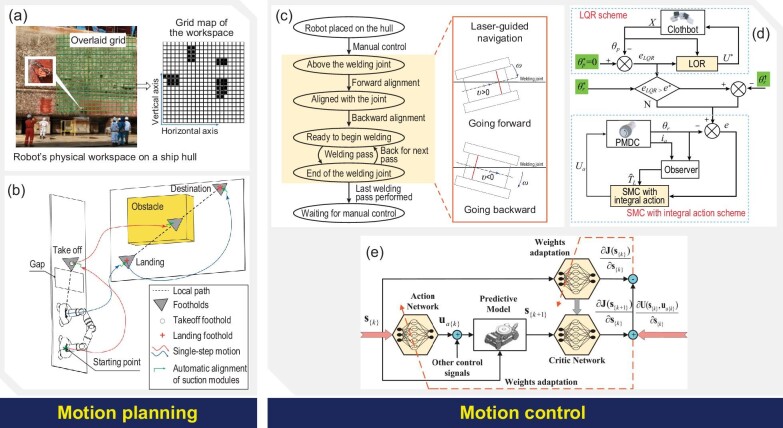
Control method for climbing robots. The typical motion planning for climbing robots can be divided into two categories: (a) motion planning for continuous locomotion, adapted with permission from Ref. [121], and (b) motion planning for intermittent locomotion, adapted with permission from Ref. [34]. Three typical motion control methods for climbing robots are (c) classical control methods, adapted with permission from Ref. [19]; (d) advanced control methods, adapted with permission from Ref. [124]; (e) artificial intelligence–based control methods, adapted with permission from Ref. [127].

Autonomous and efficient manufacturing is a core feature of climbing manufacturing robots. In view of the specificity of the climbing robot's working environment, autonomous operation can be divided into two modules: motion planning and motion control. Representative motion planning and motion control methods for the climbing robot are shown in Fig. 5.

### Motion planning

Research on motion planning for climbing robots can also be divided into two categories in accordance with the characteristics of the locomotion mechanism: motion planning for continuous and intermittent locomotion mechanisms.

Climbing robots with continuous locomotion mechanisms such as wheels and tracks have relatively mature motion models. The focus of motion planning is on the arrival of the set point and efficient coverage of the full surface, with relatively more emphasis on path planning. For point stabilization control tasks such as hole-making operations at a designated point, the path planning of a climbing robot is a common requirement in situations where different planes or obstacles exist in the working area. Common algorithms, such as RRT in path planning, have been successfully applied to climbing robots [54]. At the same time, intelligent optimization algorithms for path planning are research hotspots in this domain. For example, improved particle swarm optimization has been used to achieve efficient full-coverage path planning for a glass façade-cleaning robot [35]. Similarly, a Glasius-based bioinspired neural network path planning method was proposed to autonomously plan energy-efficient full-coverage paths in real time for ship hull inspection [121]. The adaptive coverage path planning approach based on the concepts of foraging and risk of predation in the predator–prey relationship can enable a robot to obtain full coverage of target surfaces in real time amid various unforeseen changes in the environment [122]. Current research on motion planning for climbing robots with continuous locomotion mechanisms mainly focuses on full-coverage path planning, which is closely related to climbing manufacturing robot applications, such as grinding and polishing, cleaning, and inspection.

Motion planning for climbing robots with intermittent locomotion mechanisms, such as legged, inchworm-inspired, or clamped mechanisms, is mainly focused on overcoming gravity in order to achieve stable motion on vertical surfaces. Owing to the different morphologies of intermittent locomotion mechanisms and the lack of a well-recognized motion model, motion planning largely focuses on gait planning. Gait planning of bipedal climbing robots has received the most attention. Guan *et al*. developed three gaits for an inchworm-inspired climbing robot W-Climbot: inchworm gait, swinging-around gait, and flipping-over gait [104]. The trafficability of the robot can be further improved by a hierarchical framework that divides gait planning into global route planning, local foothold planning, and single-step motion planning [34]. Similar motion planning with an inchworm-like climbing gait was also proposed for a novel cable-climbing robot called CCRobot-III [45]. The hybrid heuristic and sampling-based planning method for dynamic climbing behaviour can also be used for legged climbing robot gait planning [123]. Current motion planning for climbing robots with intermittent locomotion mechanisms primarily considers the robot's fundamental motion capabilities, such as passability and obstacle-crossing capabilities, and has not yet addressed the constraints proposed for operational tasks. In reality, climbing manufacturing robots should consider the impact of external forces, such as cutting forces, if they are aiming for machining, which is also an important research area for climbing motion planning of manufacturing robots.

### Motion control

One of the most important characteristics of climbing manufacturing robots, which are highly autonomous special robots, is their capacity for independent machining. Developing universally applicable control systems is challenging because of various adhesion and locomotion mechanisms. The existing control methods for climbing robots can be classified into three categories: classical control, advanced control and artificial intelligence.

#### Classical control methods

The goal of the current research on climbing robots is to realize the climbing function. Therefore, after completing the structural design, researchers prefer to implement the robot's basic control using conventional PID control methods. For example, the magnetic tracked robot in [19] used a laser feedback-based control law to achieve multiple passes of autonomous welding. Certain robots with special locomotion forms also use classical control laws. As in [41], a climbing robot with a dual rope and propeller uses a model-based feedforward controller to compensate for position errors due to rope deformation and slippage, and it uses a proportional-integral controller to compensate for the accumulated error due to rope dynamics. The bimodal aerial robot, consisting of a common quadrotor equipped with two passive wheels [44], uses two control methods, one of which is PID linearization control.

Classical control theory is applicable to relatively simple forms of climbing robots with a stable and easy-to-use structure, but its general performance is not satisfactory for climbing robots for machining tasks.

#### Advanced control methods

The special structure and operating environment of climbing robots endow them with special force states, making high-performance control even more challenging. Consequently, advanced control methods for different forms of climbing robots have emerged. Control of a legged climbing robot with negative pressure adsorption can be achieved using a predictor-based adaptive feedback control method [14]. Sliding mode control and model predictive control are typical advanced control methods that have been employed in some special forms of climbing robots. In a climbing robot with gripper wheels used to climb flexible clothes, a sliding mode controller with integral action along with an observer has been designed to achieve smooth robot motion [124]. For the dual–rope-driven climbing robot, rope deformation and slippage can be modelled based on the control method proposed in [41]. The position-tracking error can then be significantly reduced through feedforward compensation control based on the prediction model [125].

Accurately modelling and controlling certain special types of climbing robots is challenging because their kinematic and dynamic models exhibit nonlinear characteristics. Linearizing or decoupling the control model is a frequently used approach. For example, a feedback linearization technique was employed to control a miniature multilink climbing robot [54]. A dynamic feedback linearization strategy was developed for a quadrotor-driven wheeled climbing robot [44]. A similar multifunction robot capable of flying and climbing with multi-propellers has been stably controlled by trajectory linearization control in the legged climbing mode [126]. The quasi-decoupling control scheme for the flying climbing robot can be found in [43], which also achieved competitive performance.

The application of advanced control theory to climbing robots is distinguished by its high individuation. The complexity of popularizing applications is greatly increased by the need for distinctive control strategies for diverse robot forms. However, underpinned by increasingly powerful computing technologies, advanced control methods hold considerable promise for climbing robots. From the perspective of control performance, the robustness and accuracy of advanced control methods are attractive for manufacturing tasks such as machining and assembly.

#### Artificial intelligence–based control methods

The complex morphology of climbing robots coupled with complex force conditions makes it difficult to obtain accurate robot models. In this context, artificial intelligence methods, such as neural networks, have been applied to control climbing robots.

For the tracking control of magnetic-wheeled climbing robots, the dual-heuristic dynamic programming method can be improved by adopting random vector functional link neural networks as an actor-critic structure, which can significantly improve the transient response performance [127]. For a quadrupedal robot that mimics turtle locomotion, the antagonistic actuation model combined with an RBF compensator can be used to control the bending angle of the flippers, thus achieving more rapid tracking compared with conventional PD control [128]. In addition, neural networks can be combined with advanced control methods to achieve better performance. For example, [14] constructed adaptive neural networks to compensate for unknown dynamic disturbances and unmodelled dynamics in the adaptive control of a quadrupedal climbing robot.

Although artificial intelligence–based control methods are a hot research topic in the field of robotics, they are far from mature. Overcoming the dependence on training data and improving training quality are vital problems for their application in climbing manufacturing robots. After all, the data and time required for training are a significant luxury for manufacturing.

## DISCUSSION AND ANALYSIS

### Trend analysis for robotized intelligent manufacturing

**Figure 6.**
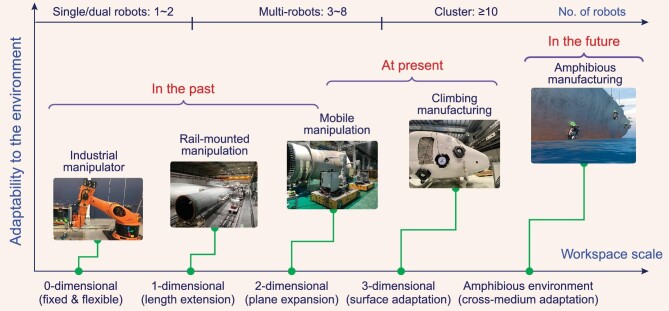
Evolution and development trend of robotized intelligent manufacturing equipment. In the last decade, robots in manufacturing have gradually developed from manipulators with fixed bases [8,10,129–131] (adapted with permission from Ref. [129]) to robots with kinematic redundancy [132], such as manipulators mounted on rails or tracks [133,134], or mobile manipulators [9,12,135,136]. At present, climbing machining robots are revolutionizing the manufacturing mode of large and complex components [50–52]. In the future, robots in manufacturing will evolve to amphibious climbing robots with cross-media capability to achieve full-field robotized manufacturing.

The development trend of robotized intelligent manufacturing is mainly reflected in three aspects, as shown in Fig. 6.

The first is the liberation of the dimensions and scale of the workspace. Robotized intelligent manufacturing equipment has gradually evolved from industrial robots with fixed bases to climbing manufacturing robots that can adsorb and move on 3D surfaces and will evolve towards amphibious climbing robot systems with mobility across water–air media to achieve full-field robotized manufacturing. At the same time, the scale of manufacturing objects that the robot can adapt to is rapidly increasing from the metre level to hundreds of metres.

The second is the improvement in adaptability to the manufacturing environment. Early robotized manufacturing equipment could be applied only to a structured environment on the factory floor. Relying on the climbing ability of vertical structures, the environmental adaptability of climbing robots has been extensively enhanced, which may even achieve *in-situ* manufacturing of equipment in field scenarios. To achieve the in-service maintenance of marine engineering equipment in the future, amphibious climbing manufacturing robots should achieve amphibious cross-media environment adaptation, truly realizing all-weather intelligent manufacturing.

The third is the enhancement of the collaborative manufacturing capability. Owing to the liberation of the workspace, climbing manufacturing robots have a more extensive physical space to achieve synergy than their predecessors. Achieving efficient, high-quality manufacturing is highly challenging for a single robot because of the expanding size of manufacturing objects, their increasingly complicated structures, and increasingly strict manufacturing quality criteria. The trend in intelligent manufacturing is the utilization of climbing manufacturing robots to create robot clusters for collaborative manufacturing.

### Challenges for climbing manufacturing robots

Table 1 summarizes the requirements imposed by various manufacturing tasks on adsorption, locomotion, positioning, and control technologies for climbing robots. Four typical machining processes are selected, namely, grinding, milling, drilling and welding; In addition, considering that intelligent manufacturing is a process that covers the entire life cycle of a product, the in-service inspection and maintenance (e.g. cleaning) during product service are also included. As can be seen from the table, the different working conditions associated with different manufacturing tasks can result in different technical demands on climbing manufacturing robots.

**Table 1.** The requirements imposed by various manufacturing tasks on different technologies for climbing robots.

Technology task	Adhesion	Locomotion	Localization	Control
Grinding and polishing	Multi-material adaptationVariable-curvature surface adaptationWithstand medium machining force (tens of N)Dusty environment adaptation	High motion efficiencyVariable-curvature surface adaptationHigh load/weight ratioLarge driving force	Superb coverageMedium positioning accuracy (mm level)Moderate reliance on external sensing	Full surface coverage motion planningMedium control precision (mm level)Highly consistent motion controlMultirobot collaboration
Milling	Multi-material adaptationVariable-curvature surface adaptationWithstand large machining force (hundreds of N)Wet and rugged environment adaptation	Medium motion efficiencyVariable-curvature surface adaptationAnchor locking capacity	Superb coverageHigh positioning accuracy (submillimetre level)Moderate reliance on external sensing	Obstacle avoidance motion planningSetpoint control with high precision (submillimetre level)
Drilling	Multi-material adaptationVariable-curvature surface adaptationWithstand large machining force (hundreds of N)	Medium motion efficiencyVariable-curvature surface adaptationAnchor locking capacity	Superb coverageHigh positioning accuracy (submillimetre level)Moderate reliance on external sensing	Obstacle avoidance motion planningSetpoint control with high precision (submillimetre level)
Welding	Multi-metal material adaptationVariable-curvature surface adaptationRugged, dusty environment adaptation	Medium motion efficiencyVariable-curvature surface adaptationHigh load/weight ratioMedium driving force	Superb coverageHigh positioning accuracy (submillimetre level)Moderate reliance on external sensing	Tracing motion planningHigh precision motion control (submillimetre level)Highly consistent motion control
In-service maintenance (cleaning)	Multi-material adaptationVariable-curvature surface adaptationWithstand medium machining force (tens of N)Wet, rugged cross-media environmental adaptation	High motion efficiencyVariable-curvature surface adaptationHigh load/weight ratioMedium driving force	Superb coverageGeneral positioning accuracy (cm level)Independent of external sensing	Full surface coverage motion planningGeneral control accuracy (cm level)Multirobot collaboration
In-service inspection	Multi-material adaptationVariable-curvature surface adaptationWet, rugged cross-media environmental adaptation	High motion efficiencyVariable-curvature surface adaptationHigh load/weight ratioMedium driving force	Superb coverageGeneral positioning accuracy (cm level)Independent of external sensing	Full coverage of motion planningGeneral control accuracy (cm level)Multirobot collaboration

Therefore, to meet the above three trends in robotized intelligent manufacturing equipment as well as the practical challenges brought by different manufacturing tasks, climbing manufacturing robots need further breakthroughs in the following aspects:

#### Adhesion principle

Adsorption is the core of climbing manufacturing robots to achieve the expansion of workspace dimensionality, along with improvement in environmental adaptability. The surfaces of large components are often free-form under hydrodynamic constraints. In addition, the adsorption module should be able to withstand dynamic loads caused by cutting forces in milling, drilling and grinding processes, and prevent the robot from desorption and slippage. In addition, the machining environment is usually dirty. Amphibious climbing manufacturing robots require adhesion abilities in both terrestrial and aquatic environments. Therefore, the innovative design of adsorption modules in multimedia environments and the seamless combination of different adsorption mechanisms are worth exploring.

#### Locomotion mechanism

Existing locomotion mechanisms have both advantages and disadvantages. The efficient wheel mechanism is difficult to adapt to rugged and slippery surfaces and lacks the ability to move across planes/surfaces. The legged mechanism with excellent terrain adaptability has poor load capacity and cannot bear the machining cutting force on vertical surfaces. The crawler structure that balances terrain adaptability and movement speed is too bulky to improve the load/weight ratio of climbing manufacturing robots. Considering the service environment of large components such as ship hulls, the motion mechanism of future climbing manufacturing robots should be lightweight with a large load/weight ratio and able to autonomously span discontinuous surfaces in amphibious environments and adapt to wet and rugged cross-media terrain under large loads.

#### Localization technology

Large-component manufacturing is shifting to an *in situ* paradigm, which means that an increasing number of large components will be manufactured in unstructured fields or even cross-media environments. While the components are increasing in size, reaching hundreds of metres, their surfaces usually lack application conditions for external measurement equipment as well as structural or textural features. Therefore, achieving localization with machining-level accuracy under the above constraints is challenging. The localization technology of climbing manufacturing robots should focus on breaking through the constraints of sparse information and the cross-media environment by extracting more sources of positioning information, overcoming the cumulative errors in large-scale positioning, and realizing cross-scale high-precision positioning without external references.

#### Control method

Conventional kinematic and dynamic models of mobile robots cannot accurately describe the motion characteristics of climbing robots on variable curvature, cross media, rugged and slippery surfaces. Coupled with strong external disturbances, control tasks exhibit strong time varying and nonlinear characteristics. Meanwhile, to cope efficiently with components of increasing sizes, forming clusters of climbing manufacturing robots is the optimal solution. However, the complexity of multirobot cooperative control increases sharply with the cluster size. To realize the autonomous assignment of manufacturing tasks and formation rearrangement with malfunctioning in cluster members, the control system of climbing manufacturing robots should set up multimodal autonomic control methods for a single robot, and a cluster dynamic information sharing network and group autonomous reconfiguration mechanism should be established.

## CONCLUSION

This paper provides a comprehensive summary of climbing manufacturing robots. First, an analysis of the evolution of robotized intelligent manufacturing clarifies that climbing manufacturing robots are an innovative way to manufacture large and complex components. The technical requirements of climbing manufacturing robots in terms of adhesion, locomotion, localization, and control were clarified through the definition of climbing manufacturing robots. Subsequently, the latest research progress on climbing robots in these four fields is reviewed, followed by research frontiers and trend discussions along with the applicability of the current research results in the manufacturing field. Finally, by looking at the development trend of robotized intelligent manufacturing equipment, the major challenges of climbing manufacturing robots in terms of adhesion principle, locomotion mechanism, localization technology, and control methods are presented.

A climbing manufacturing robot has unparalleled advantages in the *in situ* manufacturing of large components and is expected to realize a revolutionary manufacturing paradigm similar to the phenomenon of ‘ant swarm nibbles large bones’, thus laying the foundation for achieving the ambitious goal of robotized intelligent manufacturing.
